# Fostering access to and use of contextualised knowledge to support health policy-making: lessons from the Policy Information Platform in Nigeria

**DOI:** 10.1186/s12961-019-0431-4

**Published:** 2019-04-08

**Authors:** Chigozie Jesse Uneke, Etienne V. Langlois, Henry C. Uro-Chukwu, Jeremiah Chukwu, Abdul Ghaffar

**Affiliations:** 1 African Institute for Health Policy & Health Systems, Ebonyi State University, Abakaliki, Nigeria; 2 Alliance for Health Policy and Systems Research, World Health Organization, Avenue Appia 20, 1211 Geneva 27, Switzerland

**Keywords:** Policy information platform, Policy-makers, Evidence, Capacity, One-stop shop

## Abstract

**Background:**

Contextualising evidence to inform policy-making is increasingly recognised as key to developing and implementing effective health policies. Creating a one-stop shop for evidence is an approach that can facilitate timely access to the best evidence to inform policy decisions. We report outcomes after implementation of the Policy Information Platform (PIP), a pilot one-stop evidence repository in Nigeria designed to alleviate barriers to accessing policy-relevant knowledge.

**Methods:**

This cross-sectional study involved five phases, namely (1) consultation with Nigerian policy-makers to identify priority policy issues, areas of health policy information needs, and challenges and capacity constraints in accessing evidence for policy-making; (2) a stakeholder engagement workshop to formally launch the PIP; (3) extraction of data and other information from scientific articles, policy briefs, evaluation reports, grey literature and health policy documents relevant to policy-making in Nigeria (identified by Google and PubMed searches and by examination of websites of relevant Nigerian government ministries, agencies and parastatals), for use in developing the PIP website; (4) promotion of the PIP in national and state health policy meetings; and (5) evaluation of the PIP using a stakeholder survey questionnaire distributed via email and critical appraisal of the grey literature included in the PIP using the authority, accuracy, coverage, objectivity, date and significance (AACODS) checklist.

**Results:**

Priority policy areas identified by policy-makers were disease control and prevention, population health issues and health administration. Challenges identified by policy-makers were a lack of adequate capacity to access policy-relevant evidence and transform the evidence into policy. Policy-makers suggested using systematic reviews, policy briefs and rapid response mechanisms and involving policy-makers in research as ways of increasing evidence uptake for policy. A total of 126 policy-relevant, peer-reviewed scientific articles, 85 health policy documents and 201 policy-relevant grey literature documents were selected for inclusion in the PIP. Of the 195 individuals contacted via email to evaluate the PIP, 31 (15.9%) provided a response. Respondents noted that the PIP facilitated access to information based on local evidence and context-sensitive data. Barriers identified included lack of knowledge about the PIP and limited capacity of end-users to use the data compiled in the platform.

**Conclusion:**

An easily accessible one-stop shop of policy-relevant evidence can considerably improve policy-makers’ access to evidence for use in policy-making and practice.

**Electronic supplementary material:**

The online version of this article (10.1186/s12961-019-0431-4) contains supplementary material, which is available to authorized users.

## Background

Contextualising evidence to inform policy-making is increasingly recognised as key to developing and implementing effective health policies. Several previous studies have indicated that research evidence can enhance the processes of health policy development and implementation [1–5]. Despite the importance of science in health policy-making, a considerable gap remains between research evidence and the formulation and implementation of health policies, particularly in low- and middle-income countries (LMICs) [6]. Several studies have attempted to understand the suboptimal use of research evidence in policy-making, with findings suggesting the following key factors impeding evidence uptake: (1) lack of available research; (2) poor dissemination of research findings; (3) limited access to research, for various reasons including cost; (4) lack of clarity, relevance and reliability of research findings; and (5) unsuitability of the format of research output [5, 7, 8].

WHO has underlined that policy-makers need access to the right information at the right time to support evidence-informed decision-making [9]. Timely, suitably packaged and policy-relevant research can foster the use of evidence in policy processes in LMICs [10–13]. Additionally, the importance of developing concise materials and tools to communicate various types of information to policy-makers and those supporting them is well recognised [10]. Evidence exists for the value of information-packaging efforts designed to support action based on messages arising from research and other policy-relevant information [11–13]. Additional evidence suggests that effective communication relies on various factors, including readily understandable research, presentation of timely data in visually compelling formats, use of illustrative anecdotes where appropriate, creation of clear key messages about the meaning of the data, suggestions of ways to use research findings for answering important policy questions, involvement of policy-makers in the planning and execution of health research, as well as involvement of researchers in the planning and execution of health programmes, prompts of relevant articles or evidence briefs targeted to appropriate policy-makers, and establishment of relationships of trust and credibility with policy-makers [14–17].

Furthermore, the complexity of decision-making requires inputs from a broad evidence base beyond scientific research, including knowledge generated from local evidence and good practices, as well as tacit knowledge [6]. For instance, Pang [18] noted that epidemiological studies, qualitative research, experience, know-how, consensus and local knowledge should all be taken into account in health policy-making. Although the vast majority of research evidence is published in peer-reviewed journals, much contextualised and policy-relevant knowledge is confined to the grey literature and is not widely shared. The grey literature comprises a wide range of material, including policy documents, statistical publications, newsletters, fact sheets, working papers, technical reports, conference proceedings, dissertations and multimedia content [19]. There is increasing interest in the pivotal role played by the grey literature in the evidence-to-policy process [20]. For example, in May 2014, the Pisa Declaration on Policy Development for Grey Literature Resources was signed [21]. Its signatories called for increased recognition of the grey literature’s role and value by governments, academics and other stakeholders, particularly in terms of its importance for open access to research, open science, innovation, evidence-based policy and knowledge transfer [21]. Despite this interest, knowledge from the grey literature is not optimised by decision-makers, researchers and other stakeholders in the development of policies and programmes [22]. In 2012, the WHO Strategy on Health Policy and Systems Research suggested synthesising and consolidating relevant research evidence and other knowledge via country-specific national repositories of evaluations, best practices and grey literature to enable greater access to existing knowledge that could improve decision-making [22]. One of the approaches for achieving this synthesis and consolidation is the development of ‘one-stop shops’ of high-quality research evidence and policy-relevant knowledge products.

A one-stop shop for research evidence can allow health system policy-makers, stakeholders and researchers to efficiently find and use the best available research evidence in the limited time they have available to make, inform or advocate for a decision [23]. Several one-stop shops already in existence were designed to provide information related to clinical programmes and services, prescription drugs, and public health programmes and services [23–28]. In recent years, global one-stop shops have been developed to address issues related to health policy and health systems, with a focus on decision-makers, including the Health Systems Evidence repository, the Health Technology Assessment Database, EVIPNet (the Evidence to Policy Network), the Virtual Health Library and the PDQ-Evidence repository [23, 28–31]. However, to date, few one-stop shops have been established in LMICs to address their needs for health policy and health systems evidence. More specifically, there has been no health policy repository in Nigeria that could be regarded as a one-stop shop for various types of high-quality evidence (e.g. peer-reviewed research publications, expert opinion, policy documents and grey literature) specifically relevant to policy-makers’ needs for decision-making in that country.

To address this gap, the WHO Alliance for Health Policy and Systems Research and the African Institute for Health Policy & Health Systems of Ebonyi State University established a pilot repository called the Policy Information Platform (PIP) [32], to alleviate barriers to accessing the existing policy-relevant knowledge. The platform was designed to include both indexed publications and the grey literature, as well as to ensure that relevant and reliable information is available in a user-friendly format for policy and management decisions in the country. The aim of the current study was to assess the implementation of the PIP in Nigeria, with a view to documenting its functioning and policy influence, as well as understanding decision-makers’ perceptions of and satisfaction with this resource.

## Methods

This cross-sectional study involved five phases in developing the PIP, as follows: (1) consultation with policy-makers and identification of priority policy issues; (2) a stakeholder engagement workshop, with formal launch of the PIP; (3) extraction of data from policy-relevant publications and development of the PIP website; (4) promotion of the PIP website; and (5) evaluation of the PIP.

### Consultation with policy-makers and identification of priority policy issues

The PIP was planned to represent a decision-making resource and an actionable repository of knowledge, with its content designed to address key priorities identified at the national level in Nigeria. To determine the priority health policy issues to be showcased within the platform, we engaged with key policy-makers representing various areas of the health sector in Nigeria (Tables 1 and 2). These policy-makers were interviewed during face-to-face discussions or by telephone. We asked them to identify key priorities in the health policy-making process within both the government and the health sector in Nigeria, for which policy-relevant information was needed.

**Table 1:** Phases of development of the Policy Information Platform (PIP) in Nigeria

Phase	Key activities	Outcomes
Consultation with policy-makers and identification of priority policy issues	Interaction with key policy-makers representing various areas of the policy-making sector in Nigeria to identify priorities in the health policy-making process	Key policy priority areas identified by policy-makers (see Table 3)
Stakeholder engagement workshop and formal launch of PIP	Formal presentation of the PIP Nigeria website and administration of structured questionnaire to elicit information to aid in the operational effectiveness of the platform	Information generated by questionnaire: (1) areas of health policy information needs; (2) challenges and capacity constraints in accessing evidence for policy-making; (3) how evidence is utilised in the policy-making; (4) suggested ways and formats in which policy-relevant information can be made easily available and accessible to policy-makers (see Table 5)
Extraction of policy-relevant publications and development of PIP website	Publication extraction process using PubMed, Google Scholar, etc. Extracted publications classified into five main categories: (1) scientific articles, (2) policy briefs, (3) evaluation reports, (4) grey literature and (5) health policy documents	Policy-relevant publications extracted: scientific articles (*n* = 126), policy briefs (*n* = 46), evaluation reports (*n* = 23), grey literature (*n* = 201), health policy documents (*n* = 85) (see Fig. 1)
Promotion of PIP website at national and state health policy meetings	Presentation of the PIP during a stakeholder engagement event organised by West African Health Organization in Abuja in October 2015 and two state meetings in Ebonyi State in November 2015 and April 2016	Participants (*n* = 195) made aware of the existence of the PIP and given first-hand information on how to use it
Evaluation of the PIP using stakeholder survey questionnaire	Stakeholder evaluation survey undertaken via email 6 months after establishment of the PIP	Of 195 individuals contacted via email with survey questionnaire, 30 (15.4%) provided a response; respondents commended the PIP initiative and made suggestions for its improvement and sustenance

**Table 2:** Key Nigerian policy-makers consulted to identify priority health policy issues that should be addressed by the Policy Information Platform

Title	Institution	Mandate
Director of Public Health	Ministry of Health	To coordinate the formulation of public health policies and guidelines and to support their implementation and evaluation in Nigeria through health promotion, surveillance and prevention
Director of Nursing Services	Ministry of Health	To improve nursing services to patients in all public healthcare facilities
Health Systems Information Services Officer	Ministry of Health	To collect, transmit, store and manage health-related data to inform and support health management practices
Coordinator of Reproductive Health Services	Ministry of Health	To ensure improvement of maternal and newborn health, with reduction in maternal mortality ratio
Head, Department of Family Medicine	Federal Teaching Hospital, Abakaliki, Nigeria	To develop and coordinate the implementation of policies and programmes that promote the health of the family through efficient, integrated health services
Director of Primary Healthcare	Local Government Service Commission	To ensure the functioning, planning, implementation, supervision and monitoring of primary healthcare services
Chief Executive Officer	National health-based non-governmental organisation	To advocate for efficient and effective health services that will lead to improvement in health outcomes

### Stakeholder engagement workshop and formal launch of the PIP

A 1-day stakeholder engagement event was convened in September 2015 at Abakaliki, Nigeria, during which the PIP was formally launched. The purposes of the meeting were to bring together policy-makers, researchers and other stakeholders in the health sector policy-making process (including health practitioners, civil society organisations and media practitioners), to formally present the PIP Nigeria, including the website, and to elicit insights on the implementation and effectiveness of the platform to support health policy-making. A structured questionnaire (Additional file 1) was administered to participants to assess (1) their health policy-relevant information needs; (2) the challenges and capacity constraints they experienced in accessing evidence; (3) the ways in which they utilised evidence in policy-making; and (4) their suggestions of ways and formats in which policy-relevant information could be made easily available and accessible through the PIP.

### Extraction of data and other information from policy-relevant publications and development of the PIP website

The potential content of the PIP was classified into five main categories, namely (1) scientific articles, (2) policy briefs, (3) evaluation reports, (4) grey literature and (5) health policy documents. A description of the process for extracting data and other information from these publication types is provided below.

#### Scientific articles

Scientific publications reporting research done in Nigeria related to policy-making in the field of maternal, newborn and child health (MNCH) were sought. The emphasis on MNCH was a recommendation arising from the stakeholder engagement event. A PubMed search of the MEDLINE database was performed in August 2015, and studies published in English that investigated MNCH in Nigeria in relation to health policy were identified. To be included as scientific articles, these publications had to be original studies and had to contain policy recommendations. Eligible studies were retrieved and indexed in the PIP, with links to the full articles in PubMed.

#### Policy briefs

For the purpose of the PIP, a policy brief was defined as a policy document that clarifies a health policy problem, renders the evidence for addressing the problem concise and understandable, explains why the evidence is important, describes evidence-informed policy options that would be suitable actions for policy-makers to take, and provides key implementation considerations [33–35]. A Google search was performed in August 2015 with the keywords ‘policy brief’, ‘health’ and ‘Nigeria’, yielding a total of 313 entries, of which 46 policy briefs were selected. Each selected document fulfilled the following inclusion criteria: (1) must be a policy brief that meets the definition given above, (2) must focus on Nigeria, (3) must focus on the health of the population, and (4) must highlight recommendations relevant to health policy-making.

#### Evaluation reports

For the purpose of the PIP, an evaluation report was defined as a document that reports a systematic assessment of a health activity, project, programme, policy or institutional performance and that provides evidence-based information relevant to the decision-making processes, to allow an understanding of achievements or the lack thereof [36]. To identify evaluation reports, another Google search was performed in August 2015, with keywords such as ‘evaluation reports’, ‘health policy’ and ‘Nigeria’; this search yielded 363 entries, of which 23 were selected. The selected documents fulfilled the following criteria: (1) must be an evaluation report meeting the definition given above, (2) must focus on Nigeria, (3) must focus on the health of the population, and (4) must highlight recommendations relevant to health policy-making.

#### Grey literature

For the purposes of the PIP, the grey literature was defined as documents produced by all levels of government, academics, business and industry, in print or electronic formats, but not controlled by commercial publishers [19, 21]. The websites of more than 30 health-related organisations were searched for policy-relevant grey literature in August 2015. The websites were hosted by organisations such as the Nigerian health-related government ministries, departments and agencies, non-governmental organisations (NGOs), civil society organisations, and health and professional associations. The materials obtained included policy documents, statistical publications, newsletters, bulletins, fact sheets, working papers, technical reports, conference proceedings, dissertations and multimedia content. The criteria used for the selection were as follows: (1) must be a grey literature document that fulfils the definition provided above, (2) must focus on Nigeria, (3) must focus on health of the population, and (4) must be relevant to health policy-making.

#### Critical appraisal of grey literature

Each document from the grey literature was critically appraised using a modified version of the Authority, Accuracy, Coverage, Objectivity, Date and Significance (AACODS) checklist [37] (Additional file 2). This tool considers the following criteria: (1) Authority: Is the document from a reputable organisation or individual author from a reputable organisation? (2) Accuracy: What are the aims of the document? Has it been peer-reviewed or edited? Is the basis for the document clear? Is the document well structured? (3) Coverage: Are any limits clearly stated? (4) Objectivity: Does the work seem to be balanced in presentation? (5) Date: Is the date of the document given? (6) Significance: In the researcher’s estimation, will the document be of interest? The AACODS checklist has been modified by the National Institute for Clinical and Health Excellence (NICE, United Kingdom) and included among that organisation’s checklists for evidence evaluation [38]. We further refined the tool to include grading and a cut-off threshold of three points for inclusion or exclusion from the PIP. Notably, in this study, the modified AACODS checklist was used as a decision-making tool for inclusion or exclusion of grey documentation, but not to provide a scientific quality appraisal per se (e.g. low, moderate or high quality). Of 393 documents assessed, 201 (51.1%) met the threshold for inclusion.

#### Health policy documents

We searched for relevant policy documents at the websites of all Nigerian federal ministries that deal, directly or indirectly, with health, including Health and Social Services, Women Affairs and Social Development, Water Resources and Rural Development, Science and Technology, Finance and Economic Development, Environment, Education and Youth Development, and Agriculture and Natural Resources. We also searched the websites of all agencies and parastatals under the Ministry of Health, including the National Health Insurance Scheme and the National Primary Health Care Development Agency. A total of 85 health policy documents were identified and included in the PIP.

### Promotion of PIP website in national and state health policy meetings

A presentation about the PIP was made at one national and two state health policy meetings. The national meeting was a stakeholder engagement event on MNCH organised by the West African Health Organization and held in October 2015 in Abuja; 92 participants were in attendance. The two state meetings took place at Ebonyi State University in Ebonyi State, Nigeria, with 32 participants in the November 2015 event and 35 in the April 2016 meeting. Attendees at all three meetings included policy-makers, researchers and other stakeholders. The engagement process involved interaction between policy-makers and researchers on issues related to the research-to-policy interface and the establishment of formal mechanisms for continuous partnership. The PIP was introduced to the participants as a platform that would continue to provide policy-relevant information; contact information was provided so that users could request additional relevant information to address future policy-making needs.

### Evaluation of the PIP using stakeholder survey questionnaire

To assess the impact of the PIP among stakeholders who had been informed about its existence, we conducted an evaluation survey via email 6 months after the PIP was established. For this survey, 195 individuals were contacted via email. These contacts included individuals who had participated in the national and state meetings where presentations about the PIP had been made. The following questions were posed in the email survey:

1. How has the PIP Nigeria benefited or served you in your work as a policy-maker or in research as a researcher?
2. What are the drivers or motivation in your demand for information, and engagement with the PIP Nigeria website?
3. How can we make sure that the resource is known by policy-makers and researchers and used to inform the different policy steps?
4. What do you think are the facilitators and impediments to your use of the platform?
5. How can we improve the PIP Nigeria?

The written responses to the evaluation survey were analysed according to Giorgi’s phenomenological approach [39, 40]. Qualitative data analysis was conducted by assessing narratives and textual information, identifying all comments that appeared significant, abstracting units of meaning, categorising and summarising the abstractions, and returning to the extracted text to ensure a good fit.

## Results

### Outcome of consultation with policy-makers and identification of priority policy issues

The key priority areas (*n* = 17) identified by the policy-makers during the initial consultation were categorised as disease control and prevention, population health issues and health administration (Table 3). There was much emphasis on the need to prioritise policy issues regarding vulnerable populations, including pregnant women and children under 5 years of age, as well as issues relating to adolescent, reproductive and geriatric health.

**Table 3:** Health policy priority areas identified by key Nigerian policy-makers for coverage in the Policy Information Platform

Disease control and prevention	Population health issues	Health administration
(1) Infectious/communicable diseases (2) Environmental health (3) Vector control (4) Immunisation (5) Disease prevention among pregnant women and children under 5 years of age (6) Non-communicable diseases	(1) Adolescent health (2) Reproductive health (3) Gender issues in health and vulnerable populations (4) Geriatric health (5) Community participation and ownership	(1) Human resources for health (2) Primary healthcare systems (3) Leadership and governance (4) Health financing (5) National health management systems (6) Partnerships for health

### Outcome of stakeholder engagement workshop

A total of 52 participants attended the stakeholder engagement workshop and formal launch of PIP, and the profile of this group is presented in Table 4. Most participants (57.4%) were older than 45 years of age, were from the Local Government Service Commission (44%), were heads of department (39.2%) and possessed a bachelor degree (64%). Table 5 summarises the responses of participants regarding (1) areas of health policy information needs, (2) challenges and capacity constraints in accessing evidence for policy-making, (3) how evidence is used in policy-making and (4) the formats in which policy-relevant information could be made available to policy-makers (*n* = 32). Key policy information needs identified by participants pertained to adolescent health, reproductive health, health policy advocacy, programme planning and implementation, and health management information systems. Among their major capacity constraints, participants identified a lack of adequate capacity to transform evidence into policy and a lack of skill in accessing evidence and policy-relevant information. They suggested using systematic reviews, policy briefs and rapid response mechanisms, and involving policy-makers in research as ways of increasing evidence uptake for policy (Table 5).

**Table 4:** Characteristics of participants in the health stakeholder engagement event, Abakaliki, Nigeria (September 2015)

Characteristic	Number (%) of participants
Age (years)	
25–34	5 (10.6)
35–44	15 (31.9)
> 45	27 (57.4)
Total	47
Type of organisation	
Ministry of Health	10 (20.0)
Local Government Service Commission	22 (44.0)
Federal Teaching Hospital (Abakaliki, Nigeria)	9 (18.0)
Non-governmental organisation	7 (14.0)
University	2 (4.0)
Total	50
Participant designation	
Director	11 (21.6)
Head of Department	20 (39.2)
Programme Manager	6 (11.8)
Unit Officer	14 (27.4)
Total	51
Academic qualification	
Ordinary national diploma/higher national diploma	6 (12.0)
Bachelor	32 (64.0)
Masters	10 (20.0)
Doctorate	2 (4.0)
Total	50

**Table 5:** Summary of participants’ responses regarding areas of health policy information needs, capacity constraints and formats in which policy-relevant information could be made accessible

Areas of health policy information needs	Challenges and capacity constraints in accessing evidence for policy-making	How evidence is utilised in policy-making activities	Suggested ways and formats to make policy-relevant information easily available and accessible to policy-makers
(1) Public health law (2) Personnel/health administration (3) Monitoring and supervision of healthcare delivery (4) Health economics, budget and resource management (5) Health insurance (6) Adolescent health (7) Reproductive health (8) Health policy advocacy (9) Family planning (10) Programme planning and implementation (11) Primary healthcare	(1) Lack of credible, context-specific evidence (2) Government’s poor attitude towards policy-relevant evidence (3) Difficulty in finding suitable evidence (4) Inadequate format for presentation of evidence (5) Poor dissemination of evidence (6) Lack of skill in accessing evidence and policy-relevant information (7) Poor internet access at workplaces (8) Inadequate ICT knowledge and skill (9) Lack of capacity to understand evidence that is not presented plainly (10) Lack of adequate capacity to transform evidence into policy	(1) Policy dialogue (2) Proposal writing/memoranda (3) Policy formulation (4) Data analysis (5) Monthly/annual planning meetings (6) Monitoring/evaluation/impact assessment (7) Resource management (8) Development of future work plans (9) Training of staff (10) Programme implementation (11) Forecasting	(1) Systematic reviews (2) Expert information (3) Pilot studies (4) Case studies (5) National surveys (6) Monthly/annual reports (7) Journals/bulletins (8) Policy briefs (9) Executive summaries (10) Policy advocacy (11) Rapid response mechanism/timely information (12) Involvement of policy-makers in research

### Outcome of data and information extraction from policy-relevant publications for the PIP

**Fig. 1:**
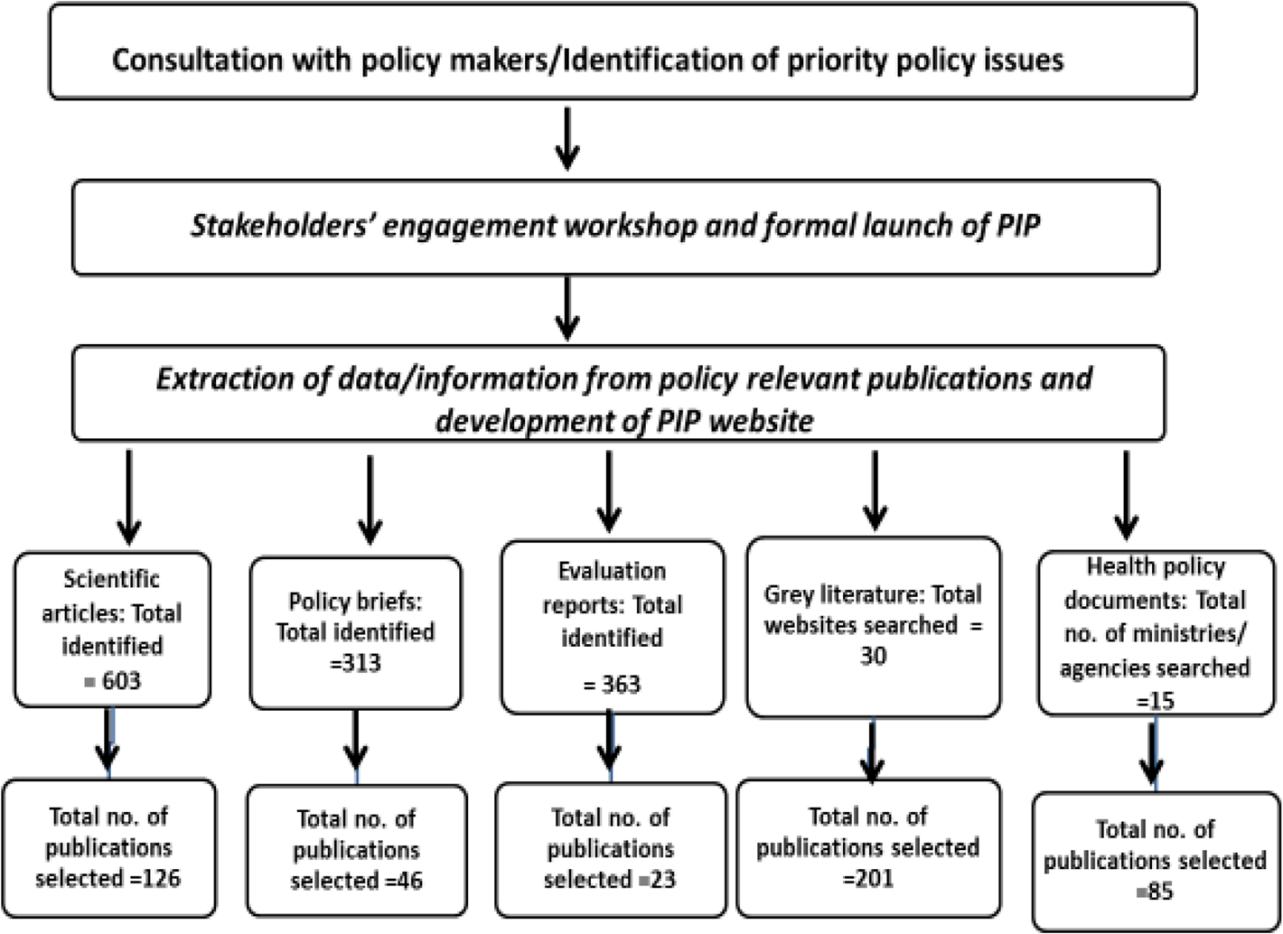
Flowchart for the process of establishing the Policy Information Platform

The extraction of data and other information from policy-relevant publications for development of the PIP was guided by the priority policy issues identified via consultation and interaction with policy-makers. Figure 1 summarises the outcome of this extraction process. A total of 126 policy-relevant peer-reviewed scientific articles, 85 health policy documents, and 201 policy-relevant grey literature documents were selected and included in the PIP.

### Outcome of PIP evaluation survey

Of the 195 individuals contacted via email with the survey questionnaire, a total of 31 provided responses, which represented a 15.9% response rate. The respondents consisted of policy-makers (22.6%), researchers (35.5%), health practitioners (35.5%) and NGO executives (6.5%). Themes that emerged from analysis of respondents’ comments are highlighted below.

#### Access and efficiency

Stakeholders highlighted the drivers of and motivation for demand for information and engagement with the PIP, including access to information and efficiency in identifying context-sensitive data.

“*What we look out for in engagement with PIP Nigeria website includes informational materials on health systems and support, health policy, health financing, advocacy and mobilisation for evidence-based and inclusive health policy and systems, links for engagement with wider networks for sustainable and resource saving systems.*” (NGO Executive)

“*The website helps to connect me to articles relevant for research, other materials and books not easy to come by. These articles and books are eye opening and very informative.*” (Researcher)

#### Policy-relevant evidence

End-users underlined the usefulness of the wide array of local evidence readily available for policy and systems decisions, and its applicability in the Nigerian health system setting.

“*The PIP Nigeria indeed served* [as] *a-one-stop-shop when seeking for policy documents on health policy. Its organisation made searching easy. The collections of local researches made discussion in relation to our Nigerian context robust.*” (Policy-maker)

#### Networking and collaboration

Stakeholders identified that a collaborative space such as the PIP can foster exchanges and inter-linkages related to evidence-informed decision-making. Researchers highlighted the importance of such platforms in catalysing dialogue with policy-makers vis-à-vis implementation research needs.

“*The Policy Information Platform PIP Nigeria has served me as a researcher in Nigeria by providing information on critical areas and issues that require research intervention and networking for optimum results. As a Research Team Leader, the PIP has also been of benefit to me and my research team by providing the platform for collaboration between researchers and policy-makers to ensure that research outputs are made readily available to policy implementers.*” (Researcher)

#### Strengthening capacities of policy-makers to use research

Policy-makers mentioned that engaging with the PIP supported their capacities to access and use research evidence in decision-making, with a particular focus on policy analysis.

“*Strengthened my capacity to apply health research in making policy decisions.*” (Policy-maker)

“*The PIP exposed me to health policies … it provided relevant resources for policy analysis … it provided scientific and evidence-based policies.*” (Policy-maker)

#### User-friendliness

Stakeholders identified the practicality and ease of navigation throughout the PIP.

“*My motivations for demand are versatility, quality, scope of studies as well as ease of use.*” (Researcher)

#### Facilitators and barriers

Respondents identified factors stimulating and impeding their use of the PIP in the context of the Nigerian health system. Facilitators included the open access feature and ongoing, iterative development and updating of content, as well as outreach features of the platform, such as prompts to users about specific evidence showcased by the PIP. Barriers included lack of knowledge about the platform and its content and limited capacities of end-users in accessing and using data included in the platform.

Regarding the facilitators, one researcher noted that “*the* we*bsite is created and maintained by University free for our use, thereby making information on global issues and updates available*”*.*

Concerning the impediments, one policy-maker stated that ignorance of the existence and availability of the platform limited access to the information that it contains.

#### Supporting demand and sustainability

Various stakeholders identified the need to advocate for the evidence and updates provided by the platform, and to make sure that the PIP is known by policy-makers and used to inform the different policy steps.

“*This would be possible by periodically engaging the stakeholders in the health sector where this platform would be introduced and its practical use made in the formulation of policy. Such programmes should be sustained.*” (Researcher)

“*The resources can be made known to people by advertising it in journals, newspapers, referring friends to the site, encouraging users to cite it in their publications, and presenting it in government/stakeholder meetings in different ministries not only the health ministry.*” (NGO executive)

“*Visibility for the resource can be assured by any or a combination of the following options, creating a link with the websites of affiliated institutions and agencies, advertise via online newsletters and blogs, writing feature articles via mass media, in-house magazines and newsletters of Ebonyi State University and affiliated organisations, etc.*” (NGO executive)

“*Do more stakeholder engagement/sensitization for them to have the knowledge of PIP, the importance and the use of the website.*” (Policy-maker)

## Discussion

### The PIP as a ‘one-stop shop’ innovation

This study represents the first attempt in Nigeria to promote a mechanism (one-stop shop) for fostering access to and use of relevant knowledge in decision-making. The PIP was designed with consideration of policy-makers as the target key users. This one-stop shop is the only platform indexing Nigerian health policy documents, policy briefs, evaluation reports and grey literature that are available online from the Ministry of Health and other related ministries, NGOs, civil society organisations and development partners. Our study suggests that the PIP platform is perceived by policy-makers and stakeholders as a useful tool to inform various steps in the policy-making process. The acceptability and reported utility of the PIP platform in Nigeria corroborates experiences in high-income health system settings, where one-stop shops have been used to ensure that policy-makers have timely access to research evidence when pressing issues emerge, which increases the prospects that research will be used in decision-making [23, 41].

The outcome of our evaluation of the PIP showed strong acceptability and feasibility of the one-stop shop platform as a resource to inform policy and systems decisions in Nigeria, including but not limited to policy analysis. Our study also identified the PIP as an asset that might strengthen the interest of policy-makers in using evidence for policy change. Decision-makers in the present study expressed their willingness to use research, which corroborates previous experiences showing that decision-makers are likely to use research evidence for policy-making when the evidence is available and accessible in a timely and user-friendly fashion [2, 4–6]. In this regard, the PIP explicitly aims to address the main barriers to integrating research into policy and practice, including high costs of access, poor clarity, relevance and reliability of research findings, and complex format of research outputs [7, 8]. Our study further reinforces previous recommendations to improve reporting formats to enhance the intelligibility of policy-relevant research outputs [2, 6, 14]. It is pertinent to state that the PIP may not be able to entirely support the translation of evidence into policy and enhance the capacity to access evidence. We are, however, confident that the availability and accessibility of the evidence in this platform are stepping stones towards enhancing any effort by policy-makers in evidence uptake and improvement of their capacities to access evidence.

### Lessons from policy-makers’ engagement

This study included active engagement of policy-makers in the process of developing the PIP platform. Interest in exchanges and engagement between researchers and policy-makers is gaining momentum worldwide, and such engagement seems better suited for the complex nature of policy-making processes than static ‘generalisable’ research (i.e. research initiated by the researchers without any interaction with policy-makers and not targeting the needs of policy-makers) [42, 43]. Interactions with policy-makers enabled the identification of key areas of policy priorities, as well as policy information needs, capacity constraints and formats in which policy-relevant information could be made available and easily accessible. The information that we obtained from the policy-makers aided in development of the platform, making it highly suitable to policy-makers’ needs and thereby facilitating the concept of ownership.

Previous evidence has suggested that, because of inadequate engagement of policy-makers, health policy needs neither drive nor determine the research priority-setting process, thereby resulting in lack of ownership of the health research agenda by policy-makers [33]. It is important for both researchers and decision-makers to recognise the value of coming together in what is in fact a symbiotic relationship in which decision-makers generate feedback from the front lines, while researchers provide expertise in research methods needed for trustworthy studies [27]. This suggests that engagement between policy-makers and researchers may ensure that knowledge generated is better aligned with the health needs of society [44, 45]. Interactions during the stakeholder engagement workshop likely led to differential use of the material in the platform, which might have increased participants’ willingness to use research or their access to the research.

Some vital lessons can be drawn from policy-makers’ involvement in the development of the PIP. One of these lessons is that policy-makers are knowledgeable about Nigerian health policy priority areas and are willing to offer helpful information to researchers. Previous studies in Nigeria have suggested that the engagement of policy-makers is crucial to the use of evidence in the planning and execution stages of health policies [17, 46]; the PIP experience corroborates this knowledge. The topics identified by policy-makers were consistent with the key health policy priority areas outlined in the most recent Nigerian National Strategic Health Development Plan (NSHDP) [47]. The NSHDP has the following eight strategic priority areas: leadership and governance for health, health service delivery, human resources for health, financing for health, national health management information system, partnerships for health, community participation and ownership, and research for health [47]. By increasing access to and usability of policy-relevant documentation, the PIP can serve as a tool to foster evidence-informed policy-making, directed towards the implementation of strategic health programmes in Nigeria, under the aegis of the NSHDP. Health policy documents, policy briefs and policy-relevant peer-reviewed articles covering these priority areas were among the publications included in the PIP.

### Value of grey literature

A major innovation of the PIP repository was the inclusion of policy-relevant grey documentation, which accounted for the largest number of publications indexed in the platform. Policy-makers specifically requested the inclusion of policy-relevant information presented in easily available and accessible formats. Specific requests pertained to publications in the form of expert information, pilot studies, case studies, national surveys, monthly and annual reports, bulletins, executive summaries and policy advocacy documents. Policy-makers thus seemed to have an understanding of and confidence in the value of grey literature as a source of vital policy-making resources and tools.

According to Lawrence et al. [48], peer-reviewed journals might be considered “*the most credible source of evidence*”, but the reality is that policy-relevant evidence is found in many kinds of resources circulating in the public sphere, most of which fall into the category of grey literature. The importance of grey literature therefore lies in its ability to communicate complex information in simple terms and to disseminate results more quickly [49]. Previous experience has shown that, in policy settings, information in the grey literature may be given greater emphasis than information from peer-reviewed journals because the language is more accessible; furthermore, more rapid and more flexible delivery of information can facilitate knowledge uptake in situations where decisions are based on competing factors (e.g. the pressures of political processes) [50–54].

It is pertinent to state that concern about the reliability of grey literature has generated some reservations. The lack of editorial control may also raise questions about the authenticity and reliability of documents in the grey literature [55]. In addition, Adams et al. [56] noted that grey literature is not bound by the publishing conventions that characterise peer-reviewed literature, and that it comes in a variety of forms, posing challenges for data management, extraction and synthesis. To address these limitations, the PIP in Nigeria employed a modified AACODS checklist to critically appraise the identified grey literature and to ensure that the platform included only documents that respect standards of scientific quality and integrity [37]. The modified AACODS checklist was used to include or exclude grey documentation from the platform, and there is a need to further study and validate the modified AACODS checklist before it can be used for traditional appraisal and grading of scientific quality. We also need additional empirical evidence through pilot testing and application of the modified and traditional AACODS approaches to study face validity and implementation determinants (e.g. appropriateness and feasibility).

### Limitations

This study had several limitations. One major limitation was our inability to critically appraise the scientific studies that were identified with PubMed and included in the PIP. Our intention is to continue to improve the quality of studies included in the PIP; to achieve this goal, we will conduct critical appraisal of scientific publications that are considered for future inclusion. Another limitation was our use of a threshold score from the AACODS checklist to determine the inclusion or exclusion of each document from the grey literature. In an earlier report, Jüni et al. [57] highlighted the problems associated with using summary scores, noting that the incorporation of quality scores as weights lack statistical or empirical justification, as previously pointed out by Detsky et al. [58]. Our intention for the future is to individually assess the relevant methodological aspects of each document to be included in the PIP, as recommended by Jüni et al. [57]. The rather low response rate to the email survey (15.9%) is yet another major limitation. Many decision-makers in Nigeria do not use email frequently, and email-based distribution is therefore the survey strategy that generates the least number of responses among policy-makers [59].

## Conclusion

The need for constant improvement and sustenance of the PIP Nigeria cannot be overstated. Therefore, we will continue to update the contents of the PIP on a monthly basis. We will also continue to expand the scope of the PIP by identifying other priority areas of interest to the policy-makers. Although the PIP focused on evidence specifically produced in Nigeria, global evidence may well be relevant to Nigerian policy-makers, as may be research from neighbouring countries. As part of our efforts to improve the reliability of the PIP, we intend to link the platform to other sources of relevant evidence for policy-makers. Potential improvements to the platform include ongoing and iterative stakeholder engagement, additional dissemination and advertising of the platform through social media and scientific conferences, and integration into the platform of a ‘frequently asked questions’ section and further user-friendly navigation guidance.

An easily accessible one-stop shop of policy-relevant evidence can considerably improve policy-makers’ willingness to use evidence in policy-making and practice. The comments of some of the policy-makers suggest that they are willing to work with researchers to improve the evidence-informed policy-making process when appropriate opportunities are made available to them. It is therefore important for researchers to initiate processes to encourage the engagement of policy-makers. The PIP is one such initiative that can facilitate interaction between policy-makers and researchers; this innovation merits further consideration.

## Additional files

Additional file 1: Policy information platform Nigeria stakeholders’ engagement workshop questionnaire. (DOCX 18 kb)

Additional file 2: The AACODS checklist for evaluation and critical appraisal of grey literature. (DOCX 22 kb)

## Abbreviations

AACODS: Authority, accuracy, coverage, objectivity, date and significance
LMICs: low- and middle-income countries
MNCH: maternal, newborn and child health
NGO: non-governmental organisation
NSHDP: National Strategic Health Development Plan
PIP: Policy Information Platform
