# Supplementary material for: Fostering access to and use of contextualised knowledge to support health policy-making: lessons from the Policy Information Platform in Nigeria
Source: Health Res Policy Syst. 2019 Apr 8;17:38. doi: 10.1186/s12961-019-0431-4 (PMC6454691; doi:10.1186/s12961-019-0431-4)
Supplement: Supplementary file 1 — Policy information platform Nigeria stakeholders’ engagement workshop questionnaire. (DOCX 18 kb) [file 12961_2019_431_MOESM1_ESM.docx]

| **POLICY INFORMATION PLATFORM NIGERIA**  ***EBONYI STATE UNIVERSITY/ALLIANCE FOR HEALTH POLICY & SYSTEMS RESEARCH***  **WORKSHOP QUESTIONNAIRE**  **Please answer the questions as correctly as possible. The questionnaire is purely for research purpose only.**  **1. Biodata**  (i). Gender Male Female  (ii). Age category 25-34 years 35-44 years ≥45 years  (iii). Marital Status Single Married Separated/Divorced  (iv). Religion Christianity Islam Others (please specify)………………….......................................  **2. Official designation attributes**  (i). Name of your organization…………………………………………………………………………........................  (ii). Designation…………………………………………………………………………………...................................  (iii). Duration in designation………………………………………………………………………...............................  (iv). Influence on policymaking process Direct Indirect  (v). Highest level of academic qualification SSCE/Diploma Bachelor Masters Doctorate  (vi). Level of operation Primary Secondary Tertiary  **3. Knowledge & application of information/communication technology**  (i). Computer literacy Yes No  (ii). If Yes, what level? Basic computer appreciation Basic computer application  (iii). Do you have a personal computer? Yes No  (iv). Do you have a computer in your office? Yes No  (v). What type(s) of operation(s) do you use the computer for?  Secretarial work   Data base management Data analysis  Others (please specify)…………………  (vi). Do you have knowledge of software usage in your job? Yes No  (vii). Do you use the internet to source for information? Yes No  (viii). If yes, how often Very frequently Frequently Occasionally  Rarely  (ix). Do you have an E-mail address? Yes No  (x). If yes, please state……………………………………………………………………………  (xi). How often do you use your E-mail address for correspondence Very frequently Frequently Occasionally Rarely Never  (xii). How would you describe your knowledge of electronic databases where health research evidence can be obtained  Grossly inadequate  Inadequate  Fairly adequate  Adequate Very adequate  (xii). How would you describe your capacity to identify and obtain relevant research evidence from electronic databases of health research  Grossly inadequate  Inadequate  Fairly adequate  Adequate Very adequate |
| --- |

| **4.** **Individual research skill** **Hints** **Ratings** | | |
| --- | --- | --- |
| (i). How would you describe your ability to summarize research results in a user-friendly way. | 1. Present research results concisely in accessible language | Grossly inadequate   Inadequate   Fairly adequate   Adequate  Very adequate |
|  | 2. Synthesize in one document relevant research as well as information and analysis from other sources. | Grossly inadequate   Inadequate   Fairly adequate   Adequate  Very adequate |
|  | 3. Link the research results to key issues and provide recommendations. | Grossly inadequate   Inadequate   Fairly adequate   Adequate  Very adequate |
| (ii). How would you describe your ability to present results of research to decision makers. | Use of charts, tables, graphs, pictogram, bullet/power point presentations, etc. | Grossly inadequate   Inadequate   Fairly adequate   Adequate  Very adequate |

| **5. As a policymaker/stakeholder in health sector, please list the areas of your health policy relevant information needs that is specific to your area of operation**  (i)...................................................................................................................................................................................  (ii)..................................................................................................................................................................................  (iii)................................................................................................................................................................................  (iv).................................................................................................................................................................................  (v)...................................................................................................................................................................................  **6. What are the challenges you experience in the assessment of evidence for policymaking?**  (i)...................................................................................................................................................................................  (ii)..................................................................................................................................................................................  (iii)................................................................................................................................................................................  (iv).................................................................................................................................................................................  (v)...................................................................................................................................................................................  **7. What are the formats of evidence that is most relevant to your job as policymaker?**  (i)...................................................................................................................................................................................  (ii)..................................................................................................................................................................................  (iii)................................................................................................................................................................................  (iv).................................................................................................................................................................................  (v)...................................................................................................................................................................................  **8. How do you utilize evidence in the policymaking process?**  (i)...................................................................................................................................................................................  (ii)..................................................................................................................................................................................  (iii)................................................................................................................................................................................  (iv).................................................................................................................................................................................  (v)...................................................................................................................................................................................  **9. How do you desire that evidence be made available to you (i.e., what format)?**  (i)...................................................................................................................................................................................  (ii)..................................................................................................................................................................................  (iii)................................................................................................................................................................................  (iv).................................................................................................................................................................................  (v)...................................................................................................................................................................................  **10. What are the various capacity constraints you have in assessing policy relevant evidence?**  ((i)...................................................................................................................................................................................  (ii)..................................................................................................................................................................................  (iii)................................................................................................................................................................................  (iv).................................................................................................................................................................................  (v)...................................................................................................................................................................................  **11. What are the main health status problems that accurate health and policy relevant information is required related to your area of operation? (Tick as many as relevant)**  Morbidity /mortality statistics,  National prevalence surveys,  Disability-adjusted life years,  Quality-adjusted life years,  Databases and systematic reviews of health research  Others please specify.....................................................................................................................................................  **12. What are the main healthcare system problems that accurate health and policy relevant information is required related to your area of operation? (Tick as many as relevant)**   Health personnel,   Health programme planning,   Programme implementation/management,   Health facilities/infrastructure,   Health care supplies  Others please specify.......................................................................................................................................................  ............................................................................................................................................................................................    **13. Please describe the process currently being used in your organization for health policymaking**  .............................................................................................................................................................................................................................................................................................................................................................................................................................................................................................................................................................................................  **14. Are there pitfalls or challenges with this process (described in No. 11 above), if yes, please outline some of them.**  (i)...................................................................................................................................................................................  (ii).................................................................................................................................................................................  (iii)...............................................................................................................................................................................  (iv)................................................................................................................................................................................  (v)..................................................................................................................................................................................  **15. Are there any kind of health research going on in your organization?** Yes No  **16. If yes to No. 15,**  (i). What research has been done or is currently undertaken?.........................................................................................  ...........................................................................................................................................................................................  (ii). What areas of research are being addressed?............................................................................................................  ..........................................................................................................................................................................................  (iii). What are the sources of funding?............................................................................................................................  .........................................................................................................................................................................................  (iv). How much money is granted to different kinds of research?..................................................................................  .............................................................................................................................................................................................  (v). Who takes the decisions concerning the fund utilization?...........................................................................................  ..........................................................................................................................................................................................  **17. Please suggest ways that policy relevant information can be made easily available and accessible to policymakers like you to encourage you to use it for policymaking**  (i)...................................................................................................................................................................................  (ii).................................................................................................................................................................................  (iii)...............................................................................................................................................................................  (iv)................................................................................................................................................................................  (v)..................................................................................................................................................................................  **18. Any further useful comment regarding what can be done to improve evidence to policy link?**  .............................................................................................................................................................................................................................................................................................................................................................................................  .............................................................................................................................................................................................................................................................................................................................................................................................  **Thank you for your response** |
| --- |
