# Supplementary material for: Fostering access to and use of contextualised knowledge to support health policy-making: lessons from the Policy Information Platform in Nigeria
Source: Health Res Policy Syst. 2019 Apr 8;17:38. doi: 10.1186/s12961-019-0431-4 (PMC6454691; doi:10.1186/s12961-019-0431-4)
Supplement: Supplementary file 2 — The AACODS checklist for evaluation and critical appraisal of grey literature. (DOCX 22 kb) [file 12961_2019_431_MOESM2_ESM.docx]

Archived at the Flinders Academic Commons: http://dspace.flinders.edu.au/dspace/

The **AACODS** checklist is designed to enable evaluation and critical appraisal of grey literature.

The Fourth International Conference on Grey Literature held in Washington, DC, in October 1999 **defined grey literature** as: "that which is produced on all levels of government, academics, business and industry in print and electronic formats, but which is not controlled by commercial publishers."

Grey literature includes theses or dissertations (reviewed by examiners who are subject specialists); conference papers (often peer-reviewed or presented by those with specialist knowledge) and various types of reports from those working in the field. All of these fall into the **“expert opinion”**

**Critical appraisal** is “the process of carefully and systematically examining research to judge its trustworthiness, and its relevance and value in a particular context” (Burls 2009)

Grey (unpublished) studies and RCTs should be appraised using the same tools as their black (published) counterparts.

| **AACODS** |  | **YES** | **NO** | **?** |
| --- | --- | --- | --- | --- |
| **Authority** | Identifying who is responsible for the intellectual content.    **Individual author:**   - Associated with a reputable organisation? - Professional qualifications or considerable experience? - Produced/published other work (grey/black) in the field? - Recognised expert, identified in other sources? - Cited by others? (use Google Scholar as a quick check) - Higher degree student under “expert” supervision?     **Organisation or group**:   - Is the organisation reputable? (e.g. W.H.O) - Is the organisation an authority in the field?     **In all cases:**   - Does the item have a detailed reference list or bibliography? |  |  |  |
| **Accuracy** | - Does the item have a clearly stated aim or brief? - Is so, is this met? - Does it have a stated methodology? - If so, is it adhered to? - Has it been peer-reviewed? - Has it been edited by a reputable authority? - Supported by authoritative, documented references or credible sources? - Is it representative of work in the field? - If No, is it a valid counterbalance? - Is any data collection explicit and appropriate for the research? - If item is secondary material (e.g. a policy brief of a technical report) refer to - the original. Is it an accurate, unbiased interpretation or analysis? |  |  |  |

Archived at the Flinders Academic Commons:

http://dspace.flinders.edu.au/dspace/

| **Coverage** | All items have parameters which define their content coverage. These limits might mean that a work refers to a particular population group, or that it excluded certain types of publication. A report could be designed to answer a particular question or be based on statistics from a particular survey.    • Are any limits clearly stated? |  |  |  |
| --- | --- | --- | --- | --- |
| **Objectivity** | It is important to identify bias, particularly if it is unstated or unacknowledged.     - Opinion, expert or otherwise, is still opinion: is the author’s standpoint clear? - Does the work seem to be balanced in presentation? |  |  |  |
| **Date** | For the item to inform your research, it needs to have a date that confirms relevance     - Does the item have a clearly stated date related to content? No easily discernible date is a strong concern. - If no date is given, but can be closely ascertained, is there a valid reason for its absence? - Check the bibliography: have key contemporary material been included? |  |  |  |
| **Significance** | This is a value judgment of the item, in the context of the relevant research area     - Is the item meaningful? (this incorporates feasibility, utility and relevance) - Does it add context? - Does it enrich or add something unique to the research? - Does it strengthen or refute a current position? - Would the research area be lesser without it? - Is it integral, representative, typical? - Does it have impact? (in the sense of influencing the work or behaviour of others) |  |  |  |

Burls, A. 2009, *What is critical appraisal*?, Bandolier, viewed 4 November 2009.

[<http://www.medicine.ox.ac.uk/bandolier/painres/download/whatis/What_is_critical_appraisal.pdf>](http://www.medicine.ox.ac.uk/bandolier/painres/download/whatis/What_is_critical_appraisal.pdf)

Jess Tyndall,

Flinders University,

Nov 2010
